# The Nature and Claims of Its Work

**Published:** 1915-06-05

**Authors:** 


### EPSOM COLLEGE AND ITS MEDICAL PENSIONERS.

### The Nature and Claims of its Work.

The Report of the Governors of Epsom College has several points of interest of which due note may be taken. The institution itself is beyond all praise and merits wide recognition by the profession. Apart from its educational value it has to be remembered that the organisation has a large benevolent activity. Thus it maintains fifty pensionerships, each of the annual value of £30, for impecunious medical men or their widows, and the same number of Foundation Scholarships for necessitous sons of medical men. This means the raising by annual contributions of about £4,500, and under the pressure of other claims there is just now a falling revenue; while at the same time the expenses, here as elsewhere, are increased as a result of war prices. The college and its benevolent activities have a strong patriotic claim on members of the profession, and it would be a matter for regret were the Governors to find themselves compelled to restrict their area of helpfulness and usefulness. Every annual subscriber, even of the modest sum of one guinea, has the opportunity of voting in the election of the annuitants. The times, it must be admitted, are not favourable for financial appeals, but we venture to think that the work of Epsom College is worthy of a wider appreciation, and that the profession will not allow an institution of such value and importance to suffer in the stress of the present crisis. The help rendered to our less fortunate brethren, or to those who survive them, is in itself a strong claim for consideration. Even still more fruitful is the educational opportunity offered to those whom fate has deprived of parental guidance and assistance. Of these latter not a few become in their turn members of the medical profession, and there are chances for the most diligent and competent in the shape of scholarships which lead in some instances to the university and in others to the larger medical schools. At the present moment there is special pride in the announcement that 350 Old Epsomians are serving with His Majesty's Forces, and that of these 250 have joined since the outbreak of the war.

It is pleasing to note that some of those who are in the best position to form a judgment have made substantial contributions to the college. This has been specially illustrated during the past year by the late Dr. Burney Yeo, who by his will left the college a sum of £3,000 as well as a substantial interest in his residuary estate, for the purpose of forming an annuity fund to be known as the "Burney Yeo Bequest." Two other legacies have been received by which further provision is made for practitioners who, as a result of illness and other misfortune, have been left in a more or less indigent position. These gifts, excellent in themselves, are valuable, too, as showing that the administration of the funds already in the hands of the Governors has attracted confidence, and that the machinery of the college, educational and benevolent, is well calculated to effect the ends for which it has been designed.

One event in the recent college history must be classed as singularly unfortunate. It seems that a lady left in her will, which was dated May 7, 1907, a sum of £500 to the "Royal Medical Benevolent Fund." Now, in its earlier days the organisation to-day known as "Epsom College" was named the "Royal Medical Benevolent College." Further, there exists another organisation, which is at present called the "Royal Medical Benevolent Fund," though until 1912 (*i.e.* more than five years after the execution of the will in question) its official style and title was the "British Medical Benevolent Fund." Hence there arose between the two organisations, each admirable in its purpose and achievement, a dispute as to which of the two was the proper recipient of the legacy. In the end the matter came into Court, and the decision of the Judge was adverse to the claim of Epsom College. The technical rules of evidence did not permit the solicitor who drafted the will to testify, as he was prepared to do, that the testatrix had expressed her intention of leaving the money to the charity to which she and her immediate relatives had been regular subscribers. As a matter of fact, it was to the institution now known as Epsom College that these subscriptions had been given, while there was no evidence to show that any such payments had been paid to the British (now Royal) Medical Benevolent Fund. This evidence, however, could not be given in Court, and the Judge ruled in favour of the institution in whose title were the words of the will, "Medical Benevolent Fund." Earlier in the trial he had said that if either of the parties knew, either by inadmissible evidence or otherwise, that the legacy was intended for the other the contest ought to be closed. Naturally this dispute between the two organisations produced bitter feelings, and from other evidence which has recently come to light it is manifest that the college has a very strong moral claim on the money. On the other hand, it is easy to understand that the committee of the "Fund" feel themselves bound as trustees by the decision of the High Court. The wonder is that the issue was ever made a matter for legal contest. It ought surely to have been easy for two bodies of men, equally anxious to act fairly and with scrupulous regard to the claims of honour, and all alike free from personal motives, to have agreed on some impartial arbitrator to whom, untrammelled by technical rules, all the evidence could have been submitted. The letting out of strife, unfortunately, is easily accomplished, and, as is not uncommon, it is doubtful whether either party has gained from the contest. If the "Fund" has secured the £500 it has received, also, whether justly or unjustly, the reputation of having acted somewhat aggressively and for its own aggrandisement, and it has been told this by one of its prominent members who is not unpractised in the art of blunt speech.

